# Calculation of Coke Layers Situation in the Cohesive Zone of Blast Furnace

**DOI:** 10.3390/ma14010192

**Published:** 2021-01-03

**Authors:** Mikolaj Bernasowski, Arkadiusz Klimczyk, Ryszard Stachura

**Affiliations:** Faculty of Metals Engineering and Industrial Computer Science, AGH University of Science and Technology, Mickiewicza 30, 30-059 Krakow, Poland; Arkadiusz.Klimczyk@agh.edu.pl (A.K.); stachura@metal.agh.edu.pl (R.S.)

**Keywords:** ironmaking, blast furnace, cohesive zone, softening, numerical modelling, horizontal probe

## Abstract

Coke is the only batch component that does not soften in blast furnace thermal conditions. It is especially important at the temperatures of the cohesive zone forming because coke layers are the only gas-permeable charge. The aim of the work described in this article is the identification of individual coke layers situation in the cohesive zone. Numerical calculations of the cohesive zone situation are based on the horizontal below burden probe measures, however, coke layers are calculated using analytical geometry. The results can be presented as a bitmap; the individual and total area of the coke layers passing gases through the cohesive zone is also calculated. This form of results allows for subjective but quick assessment of the blast furnace operation by its crew.

## 1. Introduction

Hot metal (HM) production is the most expensive step in the steel production cycle. Although the efficiency of blast furnace (BF) mostly depends on the charge materials quality [1,2], mathematical modeling of partial phenomena or the whole process can help to understand and highlight current shortcomings in BF technology [3,4].

The cohesive zone (CZ) is one of the crucial issues related to the modeling of the BF process. It is a bottleneck for the flow of reducing gases in the BF shaft, and its location and shape determine the correctness and continuity of the hot metal production process.

**Figure 1:**
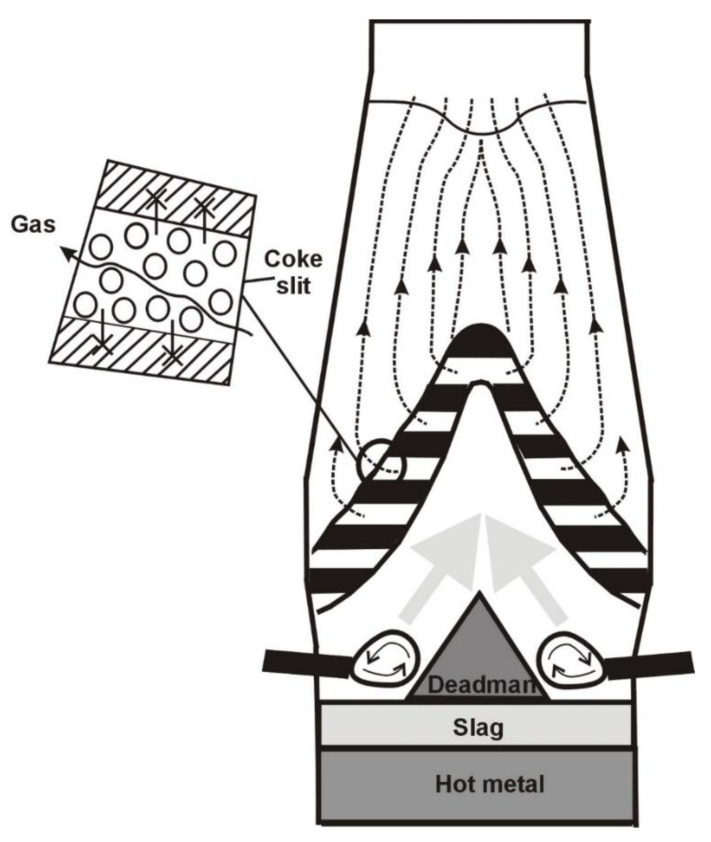
Permeability of cohesive zone in a blast furnace.

According to the definition, the cohesive zone is an area closed between the isotherms of the softening and melting of the charge oxide materials (sinter, pellets, flux, lump ore), which, being in a soft state, stick to each other and thus are impermeable to gases [5]. The only places permitting gas flow are the coke slits between cohesive ore charge layers (Figure 1). Thus, the cohesive zone plays the role of a gas distributor in the charge materials solid-state area [6].

Numerous experimental and numerical studies devoted to charging particle behavior or identifying the shape and location of the cohesion zone can be found elsewhere. Among them should be mentioned cold models [7,8,9] or mathematical models [10,11,12,13,14,15]. Also, more advanced modeling techniques like the discrete element method (DEM) [9,16,17,18], computational fluid dynamics (CFD) models [19,20,21,22,23] and a combination of the two [24,25,26,27,28] are also reported.

All the mentioned models have a common goal, namely, to describe phenomena that cannot be measured or observed directly due to the working conditions of a sealed object, which a blast furnace is. However, cold models contribute to an understanding of macro-scale particle motion phenomena, while on the other hand, DEM–CFD models help to visualize the interaction between the different phases on a micro-scale.

The miscellaneous types of input data are used as an initial condition. Most of them are measured outside the burden by utilities like thermocouples in the wall, above burden probes, radar profilometer, or throat IR camera [13,15,29,30]; cooling water temperature may also be taken into account. However, there are measuring methods known to be more intrusive into the BF working space such as descending probe (DP) and horizontal below burden gas probe (BBP).

DP is a disposable pipe that dropped on the burden surface and allows for continuous measuring of gas composition and temperature 3–5 points from the wall to the axis of BF. Although the method is accurate, it takes a long time to reach the cohesive zone level (two–three hours), so any possible response to irregularities could be delayed. On the other hand, DP helped to learn and understand the phenomena in the BF shaft.

However, BBPs became more popular and have been widely used since the 1980s. Usually, BBP is situated 3–6 m below the burden surface and measures the gas composition and its temperature at 6–10 points (depending on the BF size). A measure lasts 3–4 min per point.

It can be seen that the mentioned models [7,8,9,10,11,12,13,14,15,16,17,18,19,20,21,22,23,24,25,26,27,28,29,30] differ from each other not only in the method used but mostly in the type and place of input data collection. The less intrusive the measure, the more sophisticated the modeling methods used. However, less complex models should be sufficient for quick assessment of BF operation, but they must be based on measurements taken as close as possible to the place where a specific phenomenon occurs.

To sum up, the selection of the modeling method for a specific phenomenon is based on the type and availability of input data, but also should depend on the speed of calculations, permissible error, and ease of implementation.

The current paper presents a mathematical model based on BBP measures. The Rist principles, namely the mass and heat transfer consideration in three separate zones, preparation, reserve, and producing, are taken as fundamentals. The work data of the blast furnace No.5 in the Krakow steel plant was used for the calculations.

The current model has been gradually developed for almost four decades. Invented by Itaya et al. [10], adopted to the conditions of Polish blast furnaces by Benesch et al. [31], upgraded by Stachura et al. [32], was finally improved by us and implemented in 2018 on the blast furnace No. 5 in Krakow. Here, the latest form is presented.

## 2. Materials and Methods

### 2.1. Object of Research

The referenced blast furnace has an overall volume of 2000 m^3^ and production ability is 1.2–1.4 million tons of hot metal annually. Hearth diameter is 9.75 m, along the perimeter of which is placed 24 tuyeres. The usual hot blast (HB) volume is about 190,000 m^3^ per hour.

**Figure 2:**
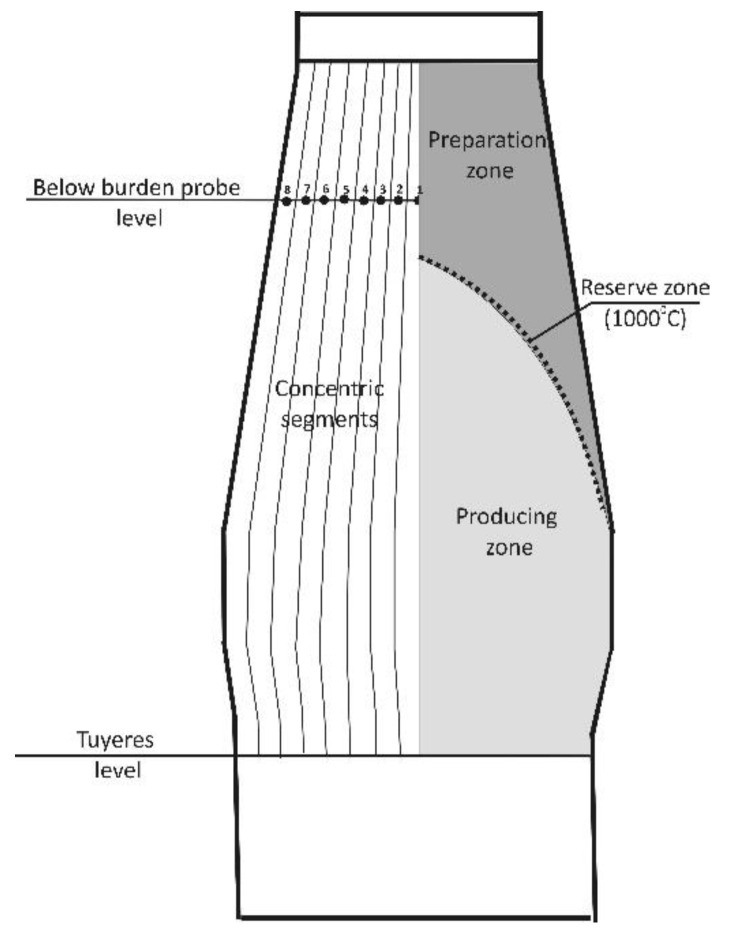
Dividing of working space according to BBP geometry and location of heat and mass exchange zones in BF.

BF No.5 is equipped with Dango & Dienenthal BBP situated 4.9 m below the throat and measures gas composition and temperature in 8 points, the distance between which is 0.54 m. In accordance with BBP geometry, a working space of BF is divided into 8 concentric segments (Figure 2). Therefore, we decided to define the levels of softening and melting isotherms occurrence for each segment and then to draw isotherms for the entire furnace. Next, it will be possible to calculate the coke slits situation. To achieve this goal, the work may be divided into the following main stages:

1. Determination of the charge materials mass and blast for each of the eight segments and their balance with the parameters of the entire furnace;
2. Determination of the preparation zone operating parameters and the levels of the 1000 °C isotherm as a thermal and chemical reserve zone;
3. Determination of the producing zone parameters using the 1000 °C isotherm level as input conditions;
4. Drawing of cohesion zone, coke layers, and calculating of their area.

### 2.2. Radial Material Balance

Radial distribution of materials charged from the top can be found using gas analyses of BBP measures. For this purpose, a system of linear equations describing the balance of elements or compounds such as nitrogen (1), hydrogen (2), carbon (3) and oxygen (4) was compiled:

(1) $$\frac{28}{22.4}\cdot N_{2}\left(k\right)\cdot 0.01\cdot V_{gas}\left(k\right)=G_{N_{2\;tuy}}+\frac{N_{2\;coke}}{C_{coke}}\cdot \left\{C_{dr}\left(k\right)+\left(G_{C_{HM}}+G_{C_{SiMnP}}\right)\cdot P_{HM}\left(k\right)\right\},$$

(2) $$\frac{2}{22.4}\cdot H_{2}\left(k\right)\cdot 0.01\cdot V_{gas}\left(k\right)+\frac{2}{18}\cdot W\left(k\right)=G_{H_{2\;tuy}}+\frac{H_{2\;coke}}{C_{coke}}\cdot \left\{C_{dr}\left(k\right)+\left(G_{C_{HM}}+G_{C_{SiMnP}}\right)\cdot P_{HM}\left(k\right)\right\},$$

(3) $$\frac{12}{22.4}\cdot \{CO_{2}\left(k\right)+CO\left(k\right)\}\cdot 0.01\cdot V_{gas}\left(k\right)=G_{C_{\;tuy}}+C_{dr}\left(k\right)+G_{C_{SiMnP}}\cdot P_{HM}\left(k\right)\cdot \frac{12}{16}Amd_{O}\left(k\right),$$

(4) $$\frac{16}{22.4}\cdot \{2CO_{2}\left(k\right)+CO\left(k\right)\}\cdot 0.01\cdot V_{gas}\left(k\right)+\frac{16}{18}\cdot W\left(k\right)=G_{O_{\;tuy}}+\left\{G_{O_{Fe}}+G_{O_{SiMnP}}\right\}\cdot P_{HM}\left(k\right)\cdot Amd_{O}\left(k\right),$$

where: (k)—index of one of eight concentric segments; CO_2_, CO, N_2_, H_2_—components of the gas measured on BBP level, in %; G_N2tuy_, G_Ctuy_, G_H2tuy_, G_Otuy_—masses of basic elements or compounds at the tuyeres level, in kg/m^3^ of the hot blast(HB); V_gas_—a volume of gas at the BBP level, in m^3^/m^3^HB; C_dr_—a mass of carbon used in solution loss reaction (direct reduction of wustite), in kg/m^3^HB; P_HM_—hot metal production, in kg/m^3^HB; W—a mass of H_2_O produced in reduction by hydrogen, in kg/m^3^HB; G_CHM_—a mass of carbon solved in hot metal, in kg/kgHM; G_CSiMnP_—a mass of carbon used for the reduction of Mn, Si, and P, kg/kgHM; N_2coke_, C_coke_, H_2coke_—contents of nitrogen, carbon, and hydrogen in coke, in mass%; G_OFe_—a mass of oxygen coming from Fe reduction, in kg/kgHM; G_OSiMnP_—mass of oxygen coming from Si, Mn and P reduction, in kg/kgHM; Amd_O_—coefficient of oxygen amendment resulting from occurring reduction processes over BBP level (will be discussed further).

Variables in bold are unknowns. The system of Equations (1)–(4) is resolved for every eight concentric segments (k). Then, it is possible to calculate the share of charge materials masses, in kg/m^3^HB:

(5) $$G_{coke}\left(k\right)=\frac{G_{CC_{tuy}}+\left(G_{C_{HM}}+G_{C_{SiMnP}}\right)\cdot P_{HM}\left(k\right)+C_{dr}\left(k\right)}{C_{coke}\cdot 0.01},$$

(6) $$G_{i}\left(k\right)=\frac{G_{i_{chg}}}{P_{HM_{chg}}}\cdot P_{HM}\left(k\right),$$

where: *i*—means any material charged from the top except coke (i.e., sinter, pellets, etc.); *G_CCtuy_*—a mass of coke carbon burnt at the tuyeres level, in kg/m^3^HB; *G_ichg_*—a mass of *i* material in a single charge, in kg/chg; P_HMchg_—hot metal production from a single charge, in kg/chg.

It can be seen that most variables are expressed per cubic meter of the hot blast, so the most important thing now is the calculation of the blast volume for particular segments. The distribution of the blast volume will depend on the geometrical dimension and porosity of each segment. In turn, the porosity index depends on the share of coke and other charge materials.

(7) $$PI\left(k\right)=PI_{chg}\cdot \frac{\frac{G_{coke}\left(k\right)}{\sum_{i=1}^{n}G_{i}\left(k\right)}}{\frac{G_{coke_{chg}}}{\sum_{i=1}^{n}G_{i_{chg}}}},$$

(8) $$V_{HB}\left(k\right)=V_{HB_{tuy}}\cdot \frac{S\left(k\right)}{\sum_{k=1}^{8}S\left(k\right)}\cdot \left(1+\frac{PI\left(k\right)-\frac{\sum_{k=1}^{8}PI\left(k\right)\cdot S\left(k\right)}{\sum_{k=1}^{8}S\left(k\right)}}{\frac{\sum_{k=1}^{8}PI\left(k\right)\cdot S\left(k\right)}{\sum_{k=1}^{8}S\left(k\right)}}\right),$$

where: *n*—max number of input material (except coke); *PI*—segmental porosity index, in m^3^/chg; *PI_chg_*—porosity index of a single charge, in m^3^/chg; *V_HB_*—segmental hot blast volume, in m^3^/h; V_HBtuy_—overall hot blast volume at the tuyeres level, in m^3^/h; S—an area of segment cross-section at the BBP level, in m^2^.

After calculating the blast distribution, the segmental material distribution in kg/chg is calculated and then their sum is compared with the real mass of the single charge. In the first iteration, the difference is high, due to the fact that the calculations were carried out to the BBP level as if reduction processes take place only below the probe. On the other hand, it is known that in such a thick charge layer above the BBP, and at such a high temperature, the mass exchange must take place. For this reason, the carbon and oxygen balance Equations (3) and (4) are amended for oxygen.

(9) $$Amd_{O}\left(k\right)=1-\frac{CO\left(k\right)\cdot t_{gas}\left(k\right)-CO_{thr}\cdot t_{thr}}{(CO_{thr}+CO_{2\;thr})\cdot 1020-CO_{thr}\cdot t_{thr}}\cdot Amd_{itr},$$

where: *CO_thr_,CO_*2* thr_* and *t_thr_*—gas composition and temperature of the gas at the throat level, in vol.%; 1020—the assumed gas temperature at the reserve zone level where charge temperature is 1000 °C (on this level CO_2_ does not exist yet and CO volume is the same as the sum of CO_thr_ and CO_2 thr_), in °C; *Amd_itr_*—iteration amendment, which during balancing of segmental masses with a real mass of single charge is changed numerically from 1 down to 0 by 0.01 for each iteration and calculations carried out in loop from Equation (1) until misbalance reaches max 2%.

### 2.3. Preparation Zone Operating Parameters

#### 2.3.1. Top Gas Temperature Radial Distribution

**Figure 3:**
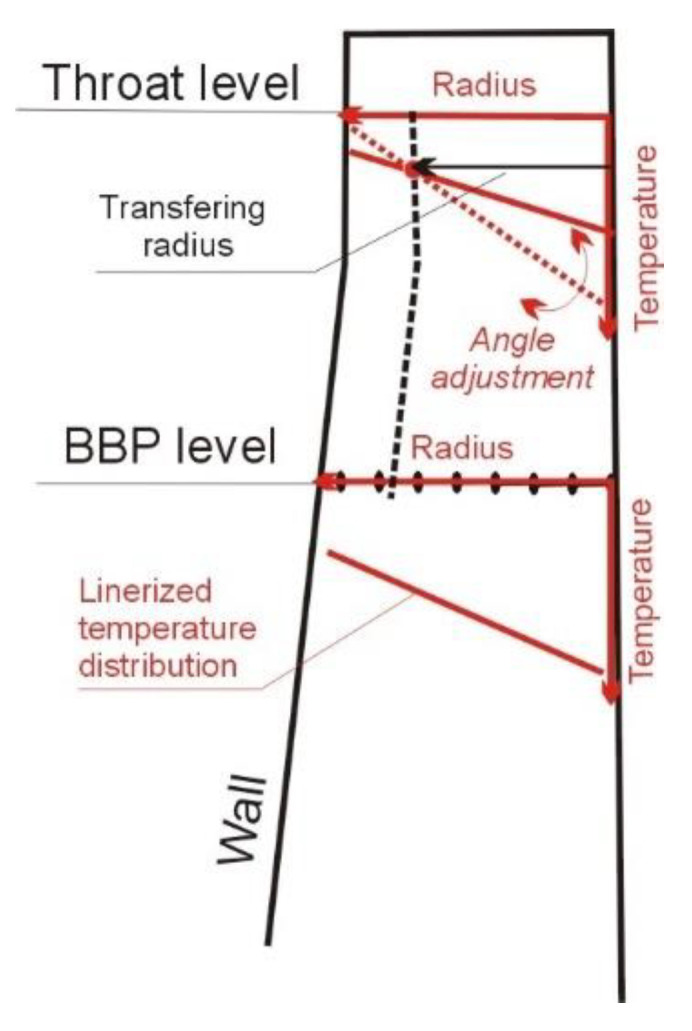
Calculation scheme of gas distribution at the throat level.

The most inconvenient for determination of the top heat exchange zone parameters is the lack of above burden probe (ABP). So, the gas temperature radial distribution at the throat level must be calculated numerically. It may be realized by transferring of the linearized temperature distribution at the BBP level to the throat level (Figure 3).

Firstly, an average gas temperature at the BBP level (10), linear dependence of the temperature distribution on the radius (11) and reference radius by which the transfer will take place are determined (12).

(10) $$t_{BBP}=\frac{\sum_{k=1}^{8}\left(V_{HB}\left(k\right)\cdot t_{gas}\left(k\right)\right)}{\sum_{k=1}^{8}V_{HB}\left(k\right)},$$

(11) $$t_{gas}\left(k\right)=A\cdot R\left(k\right)+B,$$

(12) $$R_{trsf}=\frac{t_{BBP}-B}{A},$$

where: *t_BBP_*—the average gas temperature at the BBP level, in °C; *R_BBP_*—average weighted by segments squares radius at the BBP level, in m; *R*—radius of the cylindrical segment corresponding with BBP measuring points (so R(1)=0), in m; *A,B*—linear regression coefficients; *R_trsf_*—reference radius of temperature distribution transferring, in m.

Then, the gas temperature distribution (13) and average gas temperature (14) at the throat level are calculated:

(13) $$t_{GAS}\left(k\right)=A\cdot \left\{R\left(k\right)+R_{trsf}\right\}+B-\left\{t_{BBP}-t_{thr}\right\},$$

(14) $$t_{THR}=\frac{\sum_{k=1}^{8}\left(V_{HB}\left(k\right)\cdot t_{GAS}\left(k\right)\right)}{\sum_{k=1}^{8}V_{HB}\left(k\right)},$$

where, *t_GAS_*—the gas temperature distribution at the throat level, in °C; *t_THR_*—calculated average gas temperature at the throat level, in °C.

If calculated *t_THR_* is significantly different from *t_thr_*, the *A* parameter (tangent of the linear distributed temperature angle) is changed numerically.

#### 2.3.2. Reserve Zone Situation

Preparation zone is characterized by following assumptions:

1. here is no direct reduction reaction of wustite;
2. Wall heat loss is neglected;
3. At the lower boundary, equated to the reserve zone, the batch temperature is 1000 °C and gas is 1020 °C.

Thus, heat exchange could be written as a differential equations system:

(15) $$\frac{d\{\omega_{t}\left(k\right)\cdot t_{Z}\left(k\right)\}}{dZ_{T}\left(k\right)}=K_{1}\left(k\right)\cdot S\left(k\right)\cdot \left\{t_{Z}\left(k\right)-T_{Z}\left(k\right)\right\},$$

(16) $$\frac{d\{\psi_{T}\left(k\right)\cdot T_{Z}\left(k\right)\}}{dZ_{T}\left(k\right)}=K_{1}\left(k\right)\cdot S\left(k\right)\cdot \left\{t_{Z}\left(k\right)-T_{Z}\left(k\right)\right\},$$

where:—*t_Z_* and *T_Z_* mean gas and batch temperature at the level Z respectively, in C; *ω_t_* and *ω ψ_T_*—heat capacities fluxes of gas and batch, in kJ/°C∙h; *Z_T_*—level of occurrence of T isotherm, in m; *K*_1_—volumetric heat transfer coefficient for preparation zone, in kJ/m^3^ °C∙h.

Assuming the initial integration conditions t_0_ = t_GAS_ and T_0_ = 10 °C (temperature of loading batch), the solutions of the above-mentioned equation system are:

(17) $$Z_{T}\left(k\right)=\frac{\omega_{t}\left(k\right)\cdot \alpha \left(k\right)}{K_{1}\left(k\right)\cdot S\left(k\right)\cdot \left\{\alpha \left(k\right)-1\right\}}\cdot ln\frac{\alpha \left(k\right)\cdot \left\{T_{Z}\left(k\right)-T_{0}\right\}+t_{0}\left(k\right)-T_{Z}}{t_{0}\left(k\right)-T_{0}},$$

(18) $$t_{Z}\left(k\right)=\frac{\alpha \left(k\right)\cdot \left\{t_{0}\left(k\right)-T_{0}\right\}}{\alpha \left(k\right)-1}\cdot exp\left\{\frac{K_{1}\left(k\right)\cdot S\left(k\right)\cdot Z_{T}\left(k\right)\cdot \left\{\alpha \left(k\right)-1\right\}}{\omega_{t}\left(k\right)\cdot \alpha \left(k\right)}\right\}+\frac{\alpha \left(k\right)\cdot T_{0}-t_{0}\left(k\right)}{\alpha \left(k\right)-1},$$

where: α—quotient ψ_T_/ω_t_ which is assumed to be constancy in the whole preparatory zone for each segment *k*, wherein 0 < α < 1. From Equations (17) and (18) and using known BBP level, the *K*_1_ and α values can be found by inverse method. For this purpose, it is assumed:

(19) $$Z_{T}\left(k\right)=4.91$$

(20) $$t_{Z}\left(k\right)=t_{4.91}\left(k\right)=t_{gas}\left(k\right)$$

(21) $$T_{Z}\left(k\right)=T_{4.91}\left(k\right)=\left\{1000-T_{0}\right\}\cdot \frac{t_{gas}\left(k\right)-t_{GAS}\left(k\right)}{1020-t_{GAS}\left(k\right)}+T_{0}$$

(22) $$\omega_{t}\left(k\right)=\omega_{t_{gas\left(k\right)}}\left(k\right)=V_{gas}\left(k\right)\cdot V_{HB}\left(k\right)\cdot \left\{CO\left(k\right)\cdot C_{CO}+CO_{2}\left(k\right)\cdot C_{CO_{2}}+H_{2}\left(k\right)\cdot C_{H_{2}}+N_{2}\left(k\right)\cdot C_{N_{2}}\right\}\cdot \frac{0.01}{22.4}+W\left(k\right)\cdot V_{HB}\left(k\right)\cdot \frac{C_{H_{2}O}}{18},$$

where: *C_CO_*, *C*_*CO*2_, *C*_*H*2_, *C*_*N*2_, *C*_*H2O*_—molar heat capacities of gas components calculated at the temperature of BBP level t_gas_, in kJ/°C mol.

Next, with *K*_1_ and α values, the level of 1000 °C is calculated according to Equation (17). Another value of *ω_t_* must be taken since at this level the gas temperature is 1020 °C and its composition is also different.

### 2.4. Producing Zone Operating Parameters

**Figure 4:**
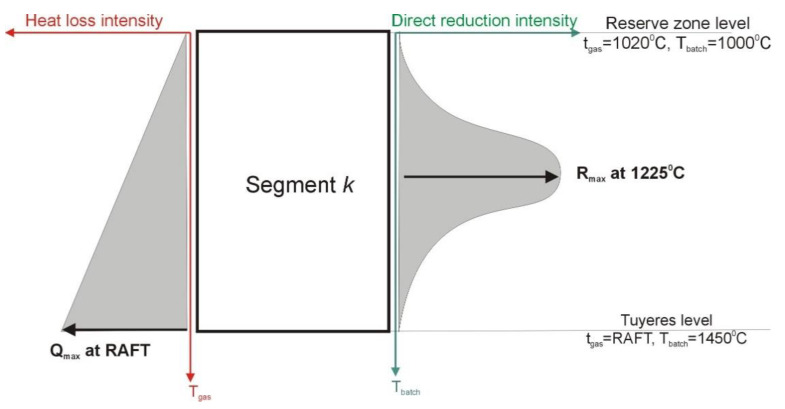
Assumptions for producing zone.

Producing zone is characterized by following assumptions (Figure 4):

1. At the top boundary (corresponding with the reserve zone level) the batch temperature is 1000 °C and gas is 1020 °C;
2. At the lower boundary the batch temperature is 1450 °C and gas is the same as raceway adiabatic flame temperature (RAFT);
3. Heat loss intensity is changed with the gas temperature, it achieves max value at the tuyeres level and min at the reserve zone level;
4. Intensity of direct reduction reaction is changed with the batch temperature according to the normal distribution; it achieves max value at 1225 °C.

In this zone, as a result of the direct FeO reduction reaction, the weight of the charge and gas is significantly decreased. Under the conditions of countercurrent motion, this phenomenon can be written as two differential equations:

(23) $$\frac{dM\left(k\right)}{dT_{Z}\left(k\right)}=-\frac{28}{12}\cdot R_{DR}\left(k\right)$$

(24) $$\frac{dm\left(k\right)}{dT_{Z}\left(k\right)}=-\frac{28}{12}\cdot R_{DR}\left(k\right)$$

where: *m* and *M* are mass fluxes of gas and batch, respectively, in kg/h; *R_DR_* is the intensity of carbon consumption by FeO direct reduction reaction in the producing zone at the temperature *T_Z_*, in kg/°C∙h. The minus sign means that gas and batch masses decrease downward.

The batch extracts heat from the gas for its heating, melting, and direct reduction, and the gas also covers heat losses. Thus, the heat transfer balance from gas to the charge can be presented in the form of the following differential equations:

(25) $$\frac{d\{\omega_{t}\left(k\right)\cdot t_{Z}\left(k\right)\}}{dZ_{T}\left(k\right)}=K_{2}\left(k\right)\cdot S\left(k\right)\cdot \left\{t_{Z}\left(k\right)-T_{Z}\left(k\right)\right\}$$

(26) $$\frac{d\{[\psi_{T}\left(k\right)+Q_{HL}\left(k\right)+R_{DR}\left(k\right)\cdot H_{DR}\left(k\right)]\cdot T_{Z}\left(k\right)\}}{dZ_{T}\left(k\right)}=K_{2}\left(k\right)\cdot S\left(k\right)\cdot \left\{t_{Z}\left(k\right)-T_{Z}\left(k\right)\right\}$$

where: *Q_HL_*—heat loss intensity at the *t_Z_* temperature, in kJ/°C∙h; *H_DR_*—enthalpy of direct reduction reaction per 1 kg of carbon, in kJ/kg; *K*_2_—volumetric heat transfer coefficient for producing zone, in kJ/m^3^·°C∙h.

Value of *Q_HL_* depends on the gas temperature and max heat loss intensity for each segment (see Figure 4):

(27) $$\frac{d\{[\psi_{T}\left(k\right)+Q_{HL}\left(k\right)+R_{DR}\left(k\right)\cdot H_{DR}\left(k\right)]\cdot T_{Z}\left(k\right)\}}{dZ_{T}\left(k\right)}=K_{2}\left(k\right)\cdot S\left(k\right)\cdot \left\{t_{Z}\left(k\right)-T_{Z}\left(k\right)\right\}$$

(28) $$Q_{max}\left(k\right)=\frac{Q_{PZ}\left(k\right)}{\int_{1020}^{RAFT}\frac{t_{Z}\left(k\right)-1020}{RAFT-1020}dt_{z}\left(k\right)}$$

where *Q_PZ_*—heat loss for the whole producing zone, in kJ/h.

However, the value of *R_DR_* depends on the batch temperature and max direct reduction intensity (see Figure 4):

(29) $$Q_{max}\left(k\right)=\frac{Q_{PZ}\left(k\right)}{\int_{1020}^{RAFT}\frac{t_{Z}\left(k\right)-1020}{RAFT-1020}dt_{z}\left(k\right)}$$

(30) $$R_{max}\left(k\right)=\frac{C_{dr}\left(k\right)\cdot V_{HB}\left(k\right)}{\int_{1000}^{1450}e^{-\frac{1}{2}{\left(\frac{T_{Z}\left(k\right)-1225}{75}\right)}^{2}}dT_{z}\left(k\right)}$$

**Figure 5:**
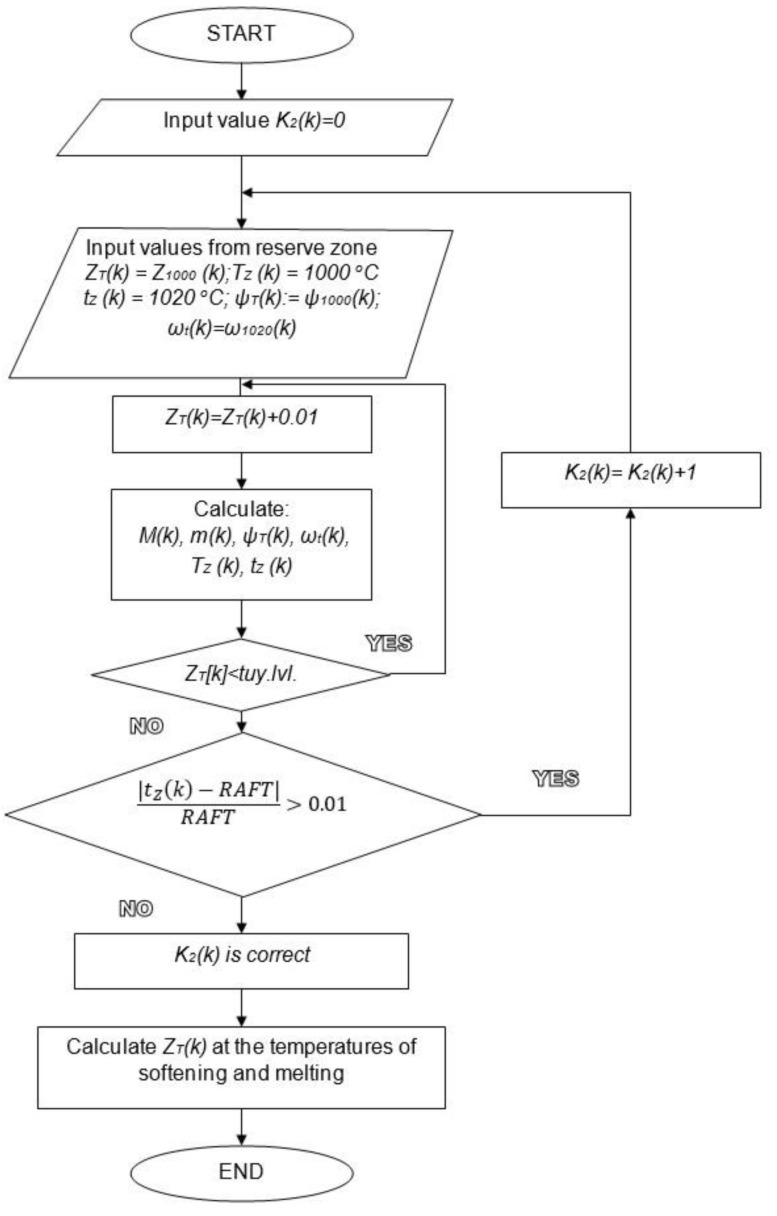
Algorithm of softening and melting isotherm levels calculations for each segment.

Producing zone calculations are conducted numerically starting from the reserve zone level to tuyeres level (25.4 m) with the step of 0.01 m. Firstly, must be found coefficient *K*_2_ for each segment. Next, the softening and melting isotherms levels are calculated (Figure 5).

The temperatures of softening and melting isotherms are calculated from empirical equation:

(31) $$T_{soft}=1094.5+6.45\cdot \frac{CaO}{SiO_{2}}-0.62\cdot FeO$$

(32) $$T_{melt}=1275+84.5\cdot \frac{CaO}{SiO_{2}}-18.7\cdot FeO$$

where: CaO, SiO_2_ and FeO–mass% of respective compounds in sinter or pellets.

Equations (31) and (32) are found by sinter and pellets softening research conducted with a Tamman furnace. The method corresponds to another research [33,34,35,36,37,38].

### 2.5. Coke Layers Situation

**Figure 6:**
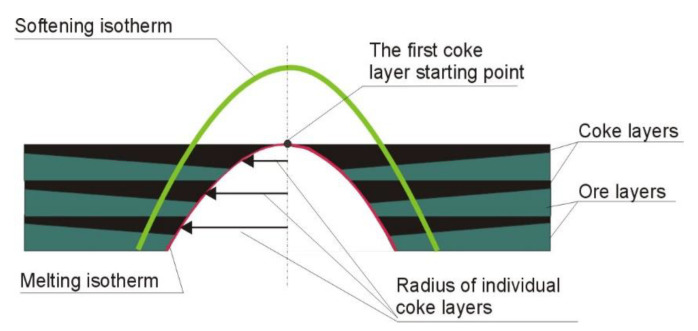
Coke layers calculation assumptions.

For the coke layers identification the following are assumed:

1. The upper boundary of the coke layer is horizontal;
2. The first coke layer in the cohesive zone always starts at the level of melting isotherm in the furnace axis (Figure 6)
3. Coke mass for each segment is reduced by carbon consumed at the direct reduction respective to the temperature of melting isotherm.

Parameters of permeable coke layers in cohesive zone can be find as following Equations (33)–(39):

(33) $$U=\frac{V_{chg}\cdot X_{chg}}{\sum_{k=1}^{8}S\left(k\right)}$$

(34) $$Z_{active}=\underset{\left(k\right)}{\max}Z_{soft}-\underset{\left(k\right)}{\min}Z_{melt},$$

(35) $$Z_{active}=\underset{\left(k\right)}{\max}Z_{soft}-\underset{\left(k\right)}{\min}Z_{melt},$$

(36) $$n_{layers}=X_{chg}\cdot t_{active}$$

(37) $$H_{coke}\left(k\right)=\frac{G_{coke-CZ}\left(k\right)}{X_{chg}\cdot S\left(k\right)\cdot 550},$$

(38) $$H_{ore}\left(k\right)=\frac{Z_{active}-H_{coke}\left(k\right)\cdot n_{layers}}{n_{layers}},$$

(39) $$S_{layer}\left(j\right)=2\cdot \pi \cdot R_{melt}\left(j\right)\cdot H_{coke}\left(k\right),$$

where: *U*—batch materials average descent speed, in m/h; *X_chg_*—number of charges loaded into the furnace within 1 h, in chg/h; *V_chg_*—bulk volume of one full charge, m^3^/chg; *Z_active_*–active (permeable) height of cohesive zone, in m; *τ_active_*—average residence time of charge materials in the active part of the cohesive zone, in h; *n_layers_*—number of coke layers, in psc; *H_coke_*—the height of coke layer, in m; *G_coke-CZ_*—coke mass lowered by carbon consumed at a direct reduction to the level of the melting isotherm, in kg/h; 550—assumed bulk density of coke, in kg/m^3^; *H_ore_*—the height of ore materials layer, in m; *S_layer_*—an area of individual coke layer, in m^2^; *R_melt_*—radius of melting isotherm at individual coke layer, in m.

## 3. Results and Discussion

Although calculations are based on the BBP measure, another data must be provided such as:

1. Charge data (mass and chemical composition of every input material);
2. Hot blast data (input volume, moisture, oxygen enrichment, temperature, PCI technology parameters);
3. Hot metal and slag chemical composition and temperature;
4. Top gas composition and temperature.

In following tables (Table 1, Table 2, Table 3, Table 4 and Table 5) are shown the main measured data and modeling results representing the work of the blast furnace after BBP entering, which took place on 24 March 2018.

Table 1 shows the radial distribution of gas parameters obtained by BBP input and average gas parameters measured at the throat level for the whole furnace.

Table 2 shows that the materials segmental distribution sum is balanced with the entire furnace at a satisfactory level. For all materials calculation error is below 1%, despite the fact that mass exchange which takes place between the BBP and throat levels is amended according to Equation (9). However, it would be better if the material distribution was calculated based on ABP data.

**Figure 7:**
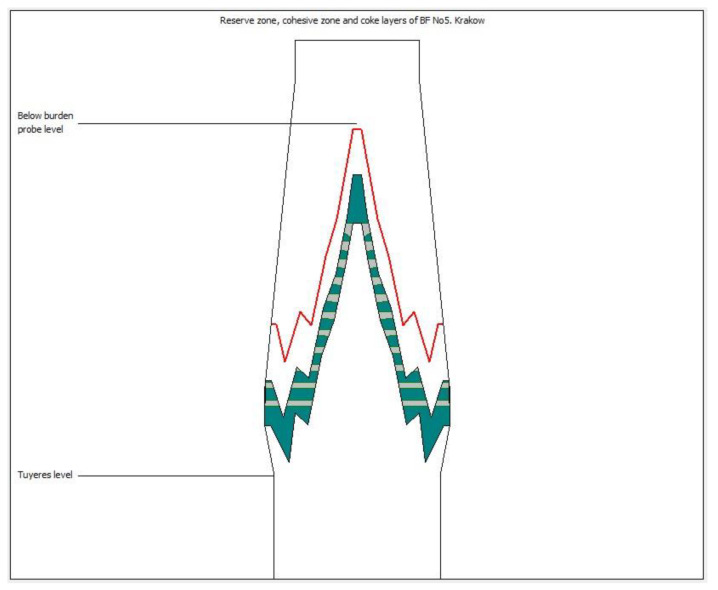
An example of cohesive zone geometry and coke layers situation.

In turn, from Table 3 it can be seen that quotient *α* in the axis region is lower than at the wall. It means that gas flux is much higher than batch flux at the axis. This is as expected since the increased axial gas flow is a prerequisite for obtaining the cohesive zone desired shape. So, the numerical calculation of *α* can be accepted as satisfactory. However, the *K*_1_ coefficient is also the result of numerical calculations, but its value is the result of closing the heat exchange balance between the throat and BBP levels. In the current model, the gas temperature radial distribution at the throat level is approximated according to Equation (13), so *K*_1_ values may be affected by a calculation error. On the other hand, from the temperature values shown in Table 1, it can be seen that heat exchange between the BBP and throat levels could not be omitted. The ideal solution for the direct determination of *K*_1_ is to use radius measurements on both levels with BBP and ABP. However, in lack of ABP the applied inverse method seems to be successful in the reserve zone level calculation. This is evidenced by the calculated gas temperature at the level of the burden isotherm 1000 °C; it is close to the assumed 1020 °C. Also from Figure 7, it can be seen that in the axis region the isotherm 1000 °C (red color line) is very close to BBP level and the measured gas temperature was 1024 °C. Calculations of other cases (see Supplementary Materials section) also show a good convergence of the mentioned parameters.

Table 4 shows the *K*_2_ coefficient values radial distribution, which is calculated as a heat exchange balance between the tuyeres and the isotherm 1000 °C levels. Because input data at these levels are known, the *K*_2_ values seem to be reliable. Calculated on the *K*_2_ basis levels of softening and melting isotherms are also in good convergence. However, in Figure 7, it can be seen that the shape of the cohesive zone (green color area) is generally the same as the shape of 1000 °C isotherm, which is assumed as the reserve zone level. It can therefore be concluded that in the present model the identification of the reserve zone level is of key importance. Figure 7 also shows the coke layer situation only in the active (permeable) part of the cohesive zone.

Summarizing the discussion, it should be noted that the current model uses BBP data as primary. However, when calculating the radial distribution of the charge and the heat transfer coefficient for the preparation zone, ABP was clearly missing and some simplifications were required. In the future, if possible, the model should be upgraded in accordance with the mentioned devices used, of which there should be at least two at different levels.

## 4. Conclusions

The paper describes the construction of a model that calculates the coke layers situation in the cohesive zone of a blast furnace; in particular, the fundamentals of mass and heat exchanges in blast furnace zones.

On the basis of measurements and chemical analyses obtained online, the model draws the situation of coke layers in the blast furnace vertical crosscut. Using only below burden probe radial data required several simplifications or assumptions in modeling. It would be much more reliable to also use the above burden probe and the profilometer. However, the presented model is satisfactory to visualize the shape of the cohesive zone and to determine the nature of the gas flow through the furnace. The model was tested in real conditions and then implemented. It can be adjusted to any blast furnace equipped with BBP only.

## Figures and Tables

**Table 1:** Gas characteristics at the BBP and throat levels.

Measurement Point	Temperature, °C	CO, vol.%	CO_2_, vol.%	H_2_, vol.%
1	1024	42.81	0.25	6.77
2	859	37.67	10.04	4.45
3	649	27.91	16.13	4.68
4	552	20.43	21.86	4.92
5	443	20.40	22.35	4.02
6	422	20.05	24.25	3.38
7	344	20.14	24.46	2.64
8	346	21.34	22.14	2.84
Throat	160	23.28	21.87	3.75

**Table 2:** Hot blast and charge material segmental distribution.

Segment Number	Hot Blast, m^3^/h	Coke, kg/chg	Sinter1, kg/chg	Sinter2, kg/chg	Pellets1, kg/chg	Pellets2, kg/chg	Coke Nut1, kg/chg	Flux1, kg/chg
1	2490	120	90	41	37	52	22	4
2	9443	553	877	399	356	506	136	39
3	16,509	869	1578	717	640	910	144	70
4	21,662	1101	2289	1041	928	1320	137	102
5	27,652	1422	3083	1402	1251	1778	177	137
6	31,988	1746	4089	1859	1659	2359	260	182
7	37,510	2055	4926	2240	1998	2841	302	220
8	47,007	2449	5463	2484	2216	3151	312	244
Sum	194,222	10,315	22,395	10,183	9084	12,916	1490	999
Furnace ^1^	194,222	10,339	22,204	10,096	9007	13,000	1498	1000
**Error,%**	0.00	0.18	0.86	0.86	0.85	0.64	0.53	0.10

^1^ Measured data.

**Table 3:** Reserve zone parameters and situation.

Segment Number	*α*, -	*K*_1_, MJ/h∙m^3^∙°C	*t_Z_*, °C	*Z*_1000_, m
1	0.6383	28.98	1020.1	5.22
2	0.6883	13.82	1020.1	6.95
3	0.7384	8.72	1020.2	10.48
4	0.7885	7.41	1020.2	12.70
5	0.8386	6.63	1020.3	16.72
6	0.8885	8.69	1020.3	15.96
7	0.9386	10.09	1020.4	18.88
8	0.9886	21.86	1020.3	16.68

**Table 4:** Producing zone parameters and cohesive zone situation.

Segment Number	*K*_2_, MJ/h∙m^3^∙°C	*Z_soft_* = *Z*_1095_, m	*Z_melt_* = *Z*_1279_, m
1	41.73	7.90	10.76
2	23.00	10.42	13.07
3	20.15	13.69	16.46
4	17.03	15.76	18.56
5	14.86	19.82	22.58
6	12.67	19.18	21.87
7	11.66	22.15	24.81
8	11.82	19.98	22.64

**Table 5:** Cohesive zone geometry.

Top Situation, m	Foot Situation, m	Active Height, m	Coke Layers Number, pcs	Total Area of Coke Layers, m^2^
7.90	24.81	11.39	11	124

## Data Availability

The data presented in this study are available in the Supplementary Materials section.

## Supplementary Materials

The following are available online at https://www.mdpi.com/1996-1944/14/1/192/s1, zipped executable application and input data files.
